# A Systematic Review and Meta-Analysis on Contrast Sensitivity in Schizophrenia

**DOI:** 10.1093/schbul/sbae194

**Published:** 2024-11-22

**Authors:** Daniel Linares, Aster Joostens, Cristina de la Malla

**Affiliations:** Vision and Control of Action Group, Departament de Cognició, Desenvolupament i Psicologia de l’Educació, Facultat de Psicologia, Universitat de Barcelona, 08035 Barcelona, Spain; Institut de Neurociències, Universitat de Barcelona, 08035 Barcelona, Spain; Vision and Control of Action Group, Departament de Cognició, Desenvolupament i Psicologia de l’Educació, Facultat de Psicologia, Universitat de Barcelona, 08035 Barcelona, Spain; Vision and Control of Action Group, Departament de Cognició, Desenvolupament i Psicologia de l’Educació, Facultat de Psicologia, Universitat de Barcelona, 08035 Barcelona, Spain; Institut de Neurociències, Universitat de Barcelona, 08035 Barcelona, Spain

**Keywords:** review, meta-analysis, contrast, sensitivity, medication, lapses, schizophrenia

## Abstract

**Background and Hypothesis:**

Understanding perceptual alterations in mental disorders can help uncover neural and computational anomalies. In schizophrenia, perceptual alterations have been reported for many visual features, including a deficit in contrast sensitivity, a key measure of visual function. The evidence supporting this deficit, however, has not been comprehensively synthesized.

**Study Design:**

We conducted a systematic review and meta-analysis of studies measuring contrast sensitivity in individuals with schizophrenia and healthy controls. Our search identified 46 studies, of which 43 focused on chronic patients.

**Study Results:**

We found that patients with chronic schizophrenia have reduced contrast sensitivity (*g* = 0.74; 95% CI, 0.55 to 0.93; *P* = 8.2 × 10^−10^). However, we found evidence that the deficit could be driven by medication. Additionally, none of the studies estimated attentional lapses, leaving it uncertain whether a potentially higher frequency of lapses in patients contributes to the observed deficit. Furthermore, only two studies comprehensively assessed visual acuity, complicating the understanding of the role of spatial frequency in the observed deficit.

**Conclusions:**

While we identified a robust deficit in contrast sensitivity among chronic schizophrenia patients, the influence of attentional lapses and medication on this impairment remains unclear. We make several suggestions for future research to clarify the underlying mechanisms contributing to this deficit.

## Introduction

People with schizophrenia not only experience visual hallucinations^1^ but also exhibit anomalies in the perception of visual stimuli.^2–4^ There are reported deficits in perceiving basic features such as contrast,^5^ color,^6^ motion^7^ and spatial frequency.^8^ There are also disturbances in the integration of information across space^9,10^ and time.^11^ Understanding deficits in visual tasks in schizophrenia could be important, as it may reveal dysfunctions in neural and computational mechanisms of the disease.^2,3,12^

One of the most reported perceptual deficits is impaired contrast sensitivity, a widely used measure to evaluate visual function that involves detecting low-contrast stimuli.^13^ Contrast is a fundamental property of visual stimuli to which the visual brain is highly responsive.^14^ Its impairment could lead to deficits in higher-level perceptual tasks such as recognizing emotions.^15^ However, the evidence supporting this deficit has not been comprehensively synthesized, leaving uncertainties about its robustness and whether it is influenced by other factors.

An area of uncertainty is the effect of medication. Most studies included patients with chronic schizophrenia taking antipsychotics. Among the limited number of studies examining the relation between medication dosage and contrast sensitivity, one reported a decrease in contrast sensitivity with medication,^16^ while the others did not find evidence supporting such an association.^15,17–26^ A few studies compared contrast sensitivity in healthy individuals with both medicated and unmedicated chronic patients, but these studies included only a small number of unmedicated patients and found conflicting results.^27–29^ Also, three studies focused on young patients who had experienced a first-episode of psychosis.^30–32^ The study including mostly medicated patients found that they had lower contrast sensitivity than controls^32^while the two including unmedicated patients found better contrast sensitivity in patients.^30,31^ Furthermore, the sensitivity enhancement observed in one of these studies transitioned to a sensitivity impairment following antipsychotic treatment.^33^ Overall, the association between antipsychotics and contrast sensitivity is not yet clear, but it might be influenced by the stage of the illness.

One factor known to influence contrast sensitivity is the spatial frequency of the stimulus—for a grating, the number of pairs of light and dark bars within one degree of visual angle.^13^ Yet, it remains unclear if the contrast sensitivity deficit in schizophrenia depends on spatial frequency. Some studies found that the deficit is more prominent at low spatial frequencies,^15,19,21,34^ suggesting a selective impairment of neurons within the magnocellular pathway,^19^ but other studies have not observed this pattern.^5,24,35,36^ A systematic review performed more than 15 years ago^37^ concluded that there was a deficit in contrast sensitivity in schizophrenia, but there was no evidence supporting selectivity for either spatial or temporal frequency (the number of oscillations in time of a stimulus whose luminance flickers). Since the publication of that review, numerous studies have studied contrast sensitivity in schizophrenia, but the evidence has not been synthesized. It is thus unclear how the spatiotemporal frequency of stimuli affects the deficit.

A deficit performing a perceptual or a cognitive task may stem from a specific impairment in the processes that the task is designed to assess, but it could also reflect a more generalized cognitive impairment, such as a lapse of attention.^3,38,39^ Lapses, which could be estimated by the proportion of errors in very easy trials,^3,38^ have not been frequently measured in studies involving patients. Recent studies, however, have shown that lapses are more prevalent in patients with schizophrenia than in healthy controls, particularly in tasks evaluating motion sensitivity,^40^ spatial suppression for contrast,^41^ spatial suppression for motion,^40^ visual acuity^42^ and working memory.^43^ One of the objectives of our study was to investigate how many studies evaluating contrast sensitivity in schizophrenia have measured lapses and whether these lapses are more common in patients than in controls.

## Results

**Figure 1.**
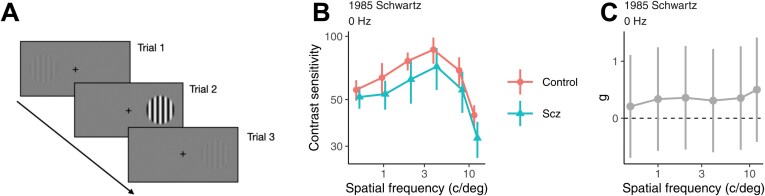
(A) Illustration of Three Trials to Measure Contrast Sensitivity. (B) Contrast Sensitivity in Individuals with Schizophrenia and Healthy Controls for Schwartz and Winstead (1985). A Small Jitter in Spatial Frequency Between Groups is Added to Minimize Overlap. Error Bars are Standard Errors of the Mean. (C) Estimated Hedges’ g for the Same Study. Error Bars are 95% Confidence Intervals

Figure 1A illustrates a typical experiment to measure contrast sensitivity using a spatial two-alternative forced-choice task.^44^ On each trial, a stimulus is displayed on one side of a fixation point and the participant reports where the stimulus was. By presenting stimuli of different contrasts on different trials, the detection threshold is estimated, which corresponds to the contrast needed to correctly report the location in a given proportion of trials. Contrast sensitivity is just the inverse of the contrast threshold.

We identified 46 studies^5,15–32,34–36,45–68^ assessing contrast sensitivity in individuals with schizophrenia and healthy controls that used independent samples (see Methods). Figure 1B shows, as an example, the results of a study^45^ that measured contrast sensitivity using gratings of various spatial frequencies presented statically (temporal frequency of 0 Hz). For each spatial frequency, we calculated the effect size, Hedges’ g, as the difference in contrast sensitivity between individuals with schizophrenia and controls divided by the pooled standard deviation, corrected for small samples (Figure 1C). This same method was applied across all studies (Supplementary Figures 1 and 2).

**Figure 2.**
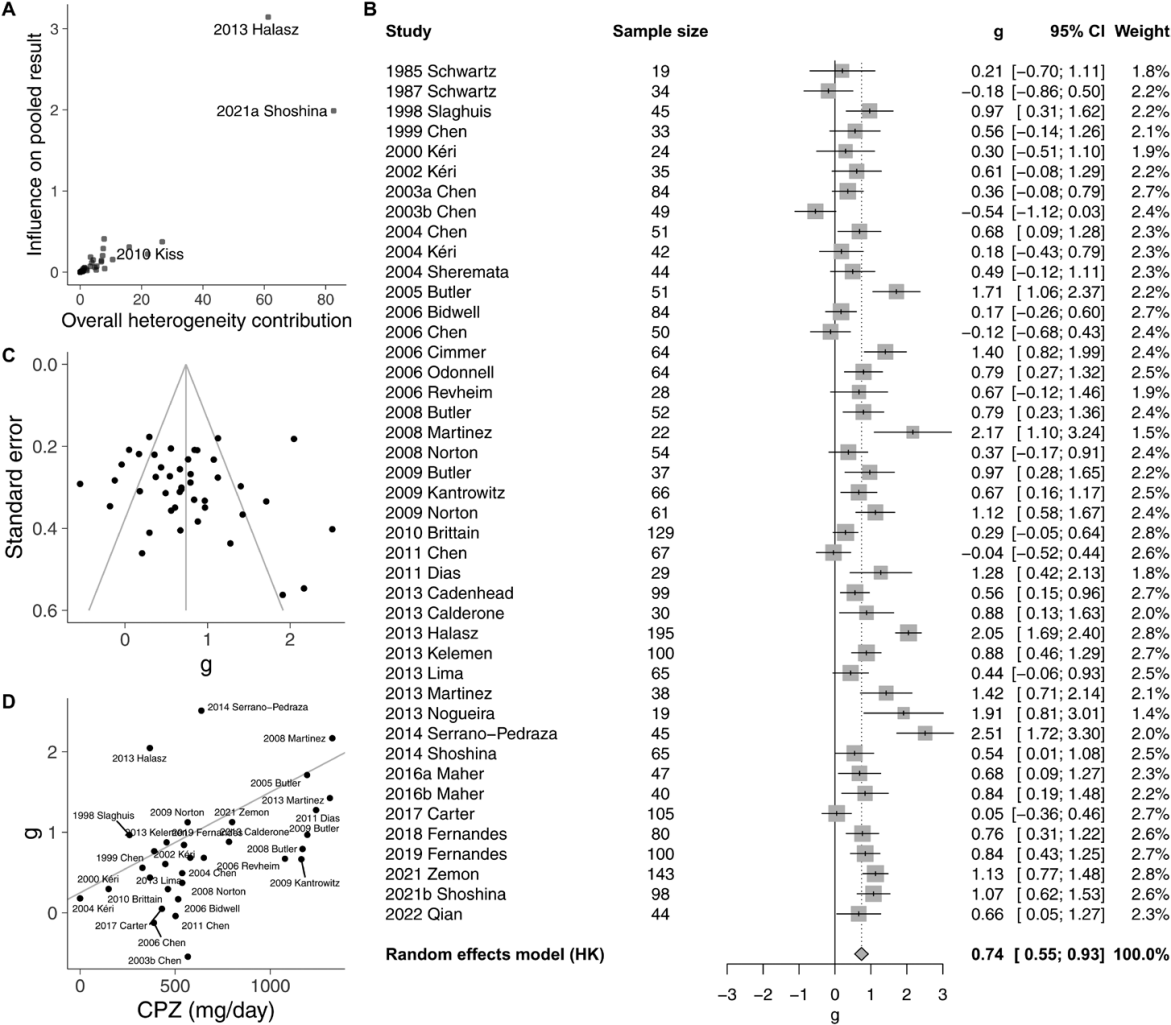
(A) Baujat Plot^69^ Showing the Contribution of Each Study to the Overall Heterogeneity as Measured by Cochran’s Q, and its Influence on the Pooled Effect Size Using a Leave-One-Out Method that Determines the Standardized Difference of the Overall Effect Size When the Study is Included Versus When it is Excluded. (B) Forest plot for the Contrast Sensitivity Deficit in Patients with Chronic Schizophrenia Including the Weight of Each Study to the Pooled Effect Size and the Sample Size. (C) Funnel Plot Indicating the Pooled Effect Size (Vertical Line) and the 95% CI for a Given Sample Size (Oblique Lines). (D) Effect of Medication on the Contrast Sensitivity Deficit. The Line Corresponds to the Regression Line

As most of the studies (31 studies) used static stimuli (temporal frequency: 0 Hz) and a low spatial frequency of 0.5 c/deg (26 studies), our first meta-analytic approach was to select one sample from each study that included those frequencies or the closest ones (Supplementary Figure 3). The pooled effect size using a random-effects model indicates a reduced contrast sensitivity amongst individuals with schizophrenia (Supplementary Figure 4; *g* = 0.64; 95% CI, 0.42 to 0.87; *P* = 7.3 × 10^−7^). There was, however, a high between-study heterogeneity (*Q*(45) = 311, *P* = 2.1 × 10^−41^; *I*^2^ = 86%; 95% CI, 82% to 89%). Figure 2A shows that the three studies contributing more to the heterogeneity were Kiss et al. (2010), Halasz et al. (2013), and Shoshina et al. (2021a). The studies by Kiss et al. (2010) and Shoshina et al. (2021a) were also the studies with a larger effect size in the opposite direction of the pooled effect size from the meta-analysis (Supplementary Figure 4). These two studies tested contrast sensitivity in young unmedicated patients who had experienced a first psychotic episode. Given that the population of first-episode patients is substantially different from the population of chronic patients,^70^ from now onwards we will not consider the studies conducted on first episodes patients, which includes these two studies and another study conducted in mostly medicated patients.^32^ We will focus, thus, on the 43 remaining studies, which evaluated patients with chronic schizophrenia.

Figure 2B shows the effect size for each study conducted in patients with chronic schizophrenia and the pooled effect size. Contrast sensitivity was reduced in individuals with schizophrenia (*g* = 0.74; 95% CI, 0.55 to 0.93; *P* = 8.2 × 10^−10^). The heterogeneity decreased compared to when we considered all the studies, but it was still very high (*Q*(42) = 195, *P* = 1.2 × 10^−21^; *I*^2^ = 79%; 95% CI, 72% to 84%). There was no evidence of publication bias (Figure 2C; Eggers’ test; intercept = 0.75; 95% CI, −1.7 to −3.2; *t* = 0.60; *P* = .55). However, the power to detect this bias might be limited due to the relatively narrow spread of precision across studies.

Across studies, there was no evidence of a difference between the mean age of individuals with chronic schizophrenia (37.7 years; range = (29.8, 47.8) years; SD = 3.52 years) and the healthy controls (36.3 years; range = (27.0, 44.1) years; SD = 4.11 years; *t*(80) = 1.6, *P* = .12), but the socioeconomic status^71^ was lower in patients (SES patients = 27.8; range = (21.5, 40.5); SD = 5.31; SES controls = 36.4; range = (20.0, 56.2); SD = 11.8; *t*(18) = 2.4, *P* = .023).

To assess the influence of medication, we considered for each study the average dosage of antipsychotics specified in chlorpromazine equivalent doses in mg/day across participants (CPZ). We found evidence that the effect size of the deficit increased with CPZ (Figure 2D, Table 1) with no evidence of a medication-independent effect given that the intercept of the regression line was not different from zero (Table 1). There was no evidence of an association of the deficit with the proportion of medicated patients (Table 1). When the three studies that tested first-episode patients were included in the meta-analysis, we also found evidence of the deficit increasing with CPZ—in this case the deficit also increased with the proportion of medicated patients (Supplementary Table 1).

**Table 1.** Effect of Different Variables on the Contrast Sensitivity Deficit

		Intercept				Slope			
variable	# samples	Estimate	95% CI	*t*	*P*	Estimate	95% CI	*t*	*P*
Using one sample from each study									
CPZ	32	0.25	(−0.21, 0.71)	1.1	.28	0.00078	(0.00015, 0.0014)	2.5	.017
Prop. medicated	43	0.075	(−0.89, 1)	0.16	.88	0.71	(−0.30, 1.7)	1.4	.16
PANSS total	27	0.087	(−2.2, 2.4)	0.080	.94	0.010	(−0.022, 0.042)	0.67	.51
Illness duration	29	0.36	(−0.44, 1.2)	0.92	.36	0.027	(−0.023, 0.076)	1.1	.28
Age	42	0.50	(−1.7, 2.7)	0.46	.64	0.0064	(−0.051, 0.064)	0.22	.82
Luminance	27	0.90	(0.38, 1.4)	3.6	.0015	−0.0015	(−0.0092, 0.0062)	−0.40	.69
Stimulus duration	34	0.68	(0.33, 1)	4	.00034	0.00021	(−5e-04, 0.00092)	0.60	.56
Stimulus size	39	1.1	(0.76, 1.5)	6.3	2.8e-07	−0.043	(−0.081, −0.0055)	−2.3	.026
Proportion females	40	1.1	(0.79, 1.5)	6.5	1.1e-07	−1.1	(−2, −0.25)	−2.6	.013
Eccentricity	41	0.58	(0.36, 0.81)	5.2	6e-06	0.16	(0.029, 0.28)	2.5	.018
Using all samples from each study									
Spatial frequency	134	0.73	(0.54, 0.92)	7.7	2.3e-12	0.014	(−0.00053, 0.028)	1.9	.059
Temporal frequency	134	0.76	(0.58, 0.95)	8	6.6e-13	0.0089	(−0.011, 0.028)	0.90	.37

Next, we examined the effect of symptom severity. Most studies evaluated symptom severity using the Brief Psychiatric Rating Scale (BPRS, Supplementary Table 1)^72^ or the Positive and Negative Syndrome Scale (PANSS, Supplementary Table 1).^73^ We converted BPRS total scores into PANSS total scores using an established method^74^ and did not find evidence that symptom severity affected the contrast sensitivity deficit (Table 1).

Given the large heterogeneity, we explored whether there were clinical variables, stimulus parameters or task parameters that moderated the effect. We did not find evidence of illness duration, age, luminance or stimulus duration influencing the contrast sensitivity deficit (Table 1). We found some evidence of the deficit decreasing with stimulus size and the proportion of females (Table 1). Next, we examined the effect of the type of task. Most studies used two-alternative forced-choice tasks (Supplementary Table 2)—seventeen the spatial version (Figure 1A) and fifteen the temporal version, where participants needed to detect whether a centrally presented stimulus was displayed in the first or second of two sequentially presented intervals (two-interval forced-choice task). A subgroup analysis indicates a higher deficit for the spatial (*g* = 0.93; 95% CI, 0.75 to 1.10) compared to the temporal version (*g* = 0.50; 95% CI, 0.18 to 0.82; *Q*(1) = 6.19, *P* = .013). Notably, the heterogeneity was remarkably lower for the spatial version (*I*^2^ = 27%) compared to the temporal version (*I*^2^ = 71%). Given that the stimuli in the spatial version were presented in the periphery and in the temporal version in the fovea, this suggests an effect of eccentricity, which was indeed evidenced when eccentricity was used as a moderator (Table 1). Regarding this exploratory analysis, it should be noted, however, that none of the eight moderators considered is statistically significant when a Bonferroni correction for multiple comparisons is applied.

Three studies reported contrast sensitivity for two stimulus durations (Kantrowitz et al., 2009; Qian et al., 2020; Zemon et al., 2021). From those studies, we selected the longer duration (500 ms) because it was closer to the most common duration used in other studies. To further assess the effect of duration, we performed an additional meta-analysis including the results for the shorter duration stimuli (approximately 30 ms). In line with the previous results, there was a deficit in contrast sensitivity (*g* = 0.64; 95% CI, 0.40 to 0.88; *P* = 3.6 × 10-6) not moderated by the duration of the stimulus (slope = 0.0003, 95% CI, −0.0006 to 0.0011; *t* = 0.57; *P* = .57).

**Figure 3.**
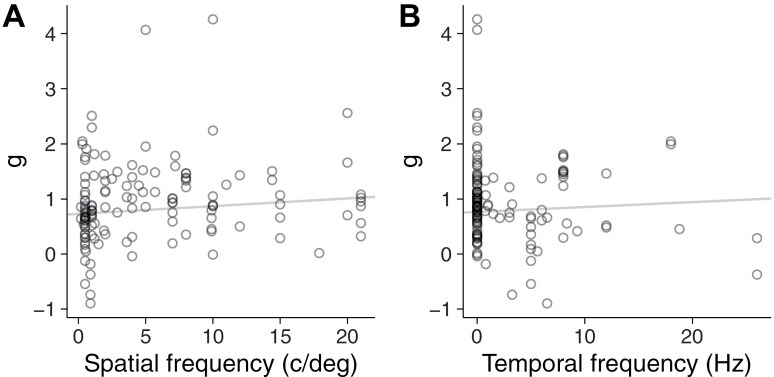
Moderating Effect of Spatial (A) and Temporal Frequency (B) in the Three-Level Random-Effects Meta-Analysis

To assess the effect of spatial and temporal frequency on the contrast sensitivity deficit, we conducted a three-level random-effects meta-analysis that incorporated the data for all the spatial and temporal frequencies from all the studies testing chronic patients (134 effect sizes). This type of analysis takes into account that within-participant measurements of contrast sensitivity at different frequencies are dependent.^75^ This analysis confirmed the large effect size for the reduction of contrast sensitivity in schizophrenia (*g* = 0.79; 95% CI, 0.61 to 0.97; *P* = 8.4 × 10^−15^). The heterogeneity variance across studies was the larger contribution to the total variance (*I*^2^_Level 3_ = 51%), but the within studies heterogeneity variance was also substantial (*I*^2^_Level 2_ = 29%). There was trend evidence of an increased effect size with spatial frequency (Figure 3A; Table 1), but no evidence of temporal frequency moderating the effect (Figure 3B; Table 1).

We also examined the effect of medication across all samples. In contrast to the single-sample meta-analysis, the increase of the deficit with CPZ was not evident (slope = 0.0002, 95% CI, −0.0003 to 0.008; *t* = 0.80; *P* = .42). To assess these apparently inconsistent results, we conducted an analysis including CPZ, spatial frequency and temporal frequency as moderators. This analysis revealed an increase in the deficit with CPZ (slope = 0.0009, 95% CI, 0.0003 to 0.0015; *t* = 3.0; *P* = .0036) and with spatial frequency (slope = 0.096, 95% CI, 0.056 to 0.14; *t* = 4.8; *P* = 6.9 × 10^−6^), but not with temporal frequency (slope = 0.032, 95% CI, −0.089 to 0.15; *t* = 4.6; *P* = .60). Moreover, we found no evidence of interaction effects, except for the interaction between CPZ and spatial frequency (slope = −0.00010, 95% CI, −0.00014 to −0.000059; *t* = 0.53; *P* = 1.3 × 10^−5^). This interaction appears to be mediated by the deficit increasing with CPZ for lower spatial frequencies and a reversed trend for high spatial frequencies (see Supplementary Figure 5). This interaction between spatial frequency and medication may explain why the single-sample meta-analysis, which predominantly included low spatial frequencies, showed an effect of medication, whereas the meta-analysis incorporating all spatial frequencies without accounting for spatial frequency did not.

A recent study suggests that studies including contrast sensitivity as a raw score might be more likely to find a greater deficit for low spatial frequencies than those that use the logarithm of contrast sensitivity to mitigate the heteroscedasticity of the data, and this might explain previous conflicting results regarding the effect of spatial frequency.^76^ We found that 13 studies used contrast sensitivity, 19 used the logarithm of the contrast sensitivity and 11 used the contrast threshold. We found no evidence of an interaction between the type of dependent variable and spatial frequency, whether considering all three types of dependent variables (*F*(2, 128) = 0.17, *P* = .84) or when focusing on contrast sensitivity and the logarithm of contrast sensitivity (*F*(1, 117) = 0.39, *P* = .53). However, a limitation of this analysis is that as we did not have access to the raw data, we were unable to transform the dependent variable for each study. Consequently, we just relied on the dependent variable used by each research group, which could potentially be associated with various aspects of their experimental designs.

Regarding the evaluation of lapses, we found that 40 studies used standard adaptive methods (Supplementary Table 2). In these methods, the contrast of the stimulus is adjusted on each trial depending on the participant’s previous responses to ensure that it remains close to the threshold value.^44^ Consequently, these methods include only a limited number of high-contrast (easy) trials, preventing the estimation of the lapse rate. Four studies (Supplementary Table 2) used the method of constant stimuli,^28,32,61,62^ which typically include high-contrast trials, but the lapse rate was not estimated. Additionally, two studies^35,45^ used the method of limits (Supplementary Table 2), a rarely used method today due to known biases.^44^

Finally, we considered the role of visual acuity. People with schizophrenia exhibit lower visual acuity than healthy controls, a finding that could be partially due to poorer correction of refractive errors.^77^ Since even minor refractive errors impair contrast sensitivity at high frequencies,^78,79^ it is possible that part of the lower contrast sensitivity at high frequencies in schizophrenia stems from diminished visual acuity.^79^ We found that most studies included only participants with normal or corrected-to-normal visual acuity, but typically setting the inclusion criterion at 20/30 (Supplementary Table 2), which is not considered a very high acuity.^79^ Only two studies reported and compared visual acuity across groups.^36,55^ In line with the broader population-level observations, Zemon and colleagues^36^ found lower visual acuity in patients with schizophrenia compared to controls. In contrast, Brittain and colleagues^55^ found similar visual acuity across groups.

## Discussion

Our meta-analysis indicates that individuals with chronic schizophrenia have lower contrast sensitivity than healthy controls and that the magnitude of the deficit is large.

We found evidence that the deficit in contrast sensitivity increased with antipsychotic dosage. This evidence is primarily derived from stimuli with low spatial frequencies, which have been the stimuli more commonly used to assess contrast sensitivity in schizophrenia. This association may result from the direct effects of the medication given that antipsychotics exert a dopamine-blocking action, and reduced dopamine levels are known to impair contrast sensitivity.^80^ Alternatively, it may reflect that more severely affected patients need higher medication doses. We did not find an association between symptom severity and the deficit. However, it remains possible that antipsychotic treatment alleviates symptom severity without significantly altering deficits in contrast perception. To better understand the role of antipsychotics in contrast sensitivity, it would be valuable to conduct studies with relatively large sample sizes that include both medicated and unmedicated patients, as well as healthy controls.

Three studies have assessed contrast sensitivity in first-episode patients.^30–32^ Notably, the two studies conducted on unmedicated patients found that they had an *enhanced* contrast sensitivity,^30,31^ an effect that cannot be attributed to a generalized cognitive impairment. This enhancement could be a promising marker for specific stages of the disease. Future research including unmedicated first-episode and chronic patients would help determine the robustness of the observed sensitivity boost and its dependence on the stage of the illness.

We found that most studies did not compare visual acuity between patients and controls, leaving it unclear whether the groups were matched in this respect. This holds importance because people with schizophrenia tend to have lower visual acuity,^77^ and reduced visual acuity decreases contrast sensitivity at high frequencies.^78,79^ While it is unlikely that a possible impairment in visual acuity explains the robust deficit observed at low spatial frequencies, it may contribute to the increase in contrast sensitivity with spatial frequency. To clarify the spatial frequency tuning of the deficit and its possible link to a magnocellular system impairment,^19,36^ future studies should directly measure visual acuity, and account for it in their analysis,^36,76^ rather than relying solely on inclusion criteria. They also should consider the use of the logarithm of the contrast sensitivity as a dependent variable in case it is necessary to mitigate the heteroscedasticity of the data—not doing so might amplify the relative contribution of low spatial frequencies to the deficit.^76^

We found that none of the studies estimated lapses, leaving it unclear whether patients with schizophrenia had more lapses than controls while performing contrast sensitivity tasks. However, this seems likely given recent findings showing that patients with schizophrenia tend to have more lapses in other perceptual tasks.^40–42^ Therefore, the possibility that the reduced contrast sensitivity observed in patients may be partially or even entirely due to a higher frequency of lapses remains unexplored,^3,38,39^ making it uncertain how much of the observed deficit is genuinely perceptual.

Future studies could estimate lapses by measuring the proportion errors in very easy trials, such as Trial 2 in Figure 1A—an approach previously used in other perceptual tasks conducted in patients with schizophrenia.^40–42^ Inattentive participants would make a relatively high number of errors on those trials. To estimate contrast sensitivity while accounting for lapses, one approach could be to exclude participants with high lapse rates from the analysis.^41^ However, a limitation of this method is that it risks excluding patients with more severe illness, who may exhibit the strongest perceptual alterations.^41^ A better alternative would be to include most participants and fit a psychometric function to each one that incorporates a lapse rate parameter—the asymptotic proportion of correct responses at high-contrast levels.^40,81–83^ Importantly, sensitivity should not be calculated as the inverse of the threshold, as the threshold is not independent of the lapse rate.^84^ Instead, sensitivity should be estimated as the inverse of the location parameter of the sigmoidal function incorporated in the psychometric function model, which is independent of lapses.^40,84,85^ Additionally, psychometric function models can incorporate a bias parameter to account for a participant’s tendency to respond to a specific location or interval.^85^

To address the substantial heterogeneity that we observed, we conducted an exploratory analysis that considered several potential moderators. There was some evidence indicating that the deficit was more pronounced when stimuli appeared at different peripheral locations. This finding aligns with previous research showing that individuals with schizophrenia have greater difficulty to distribute attention across wide spatial areas.^38^ We also hypothesize that a possible source of variability across studies could stem from differences in how instructions emphasize the need to keep focus and from the methodologies employed by experimenters to supervise participants while performing the task. Such factors could impact participants’ propensity to have lapses. Accounting for these lapses might reduce the observed heterogeneity.

Assessing contrast perception in schizophrenia is important as it may reflect a deficit in glutamatergic neurotransmission,^2,19^ which is considered to play a central role in the pathogenesis of the disease.^86^ Animal studies have shown that blocking NMDA glutamate receptors reduces neuronal response in visual areas,^87,88^ suggesting that the reduced contrast sensitivity and the reduced electrophysiological response to contrast^15,19,58,89,90^ in schizophrenia could be indicative of glutamatergic hypofunction.^2,19^ Moreover, NMDA receptors blockade has a more pronounced effect on small stimuli,^88^ which may be related to our preliminary observation that the contrast sensitivity deficit decreases with stimulus size. However, since the neural response to contrast is also modulated by other neurotransmitters, including dopamine (Silverstein & Rosen, 2015), it is crucial to clarify the role of antipsychotics in order to fully understand the contribution of the glutamatergic system to alteration in contrast perception in schizophrenia.

A limitation of our study is that we did not pre-register the study. However, our hypotheses regarding the effects of medication and spatial frequencies are well-established in the field, while the potential impact of other moderators was described as exploratory. Additionally, we think that our inclusion criteria for studies were naturally defined: we focused on studies involving patients diagnosed with schizophrenia and a control group performing a contrast sensitivity task, which is a standard procedure in the literature. As a result, the selection of studies was straightforward, with very few borderline cases.

Extensive research into the neural and computational mechanisms of perception makes the study of perceptual phenomena in schizophrenia and other mental diseases a promising venue for uncovering mechanistic anomalies. Our findings indicate that the contrast sensitivity deficit in chronic patients is a robust effect with a large effect size, which could potentially advance this pursuit. However, it is important to reassess contrast sensitivity with the appropriate controls to determine whether patients experience more lapses than controls and to assess the extent to which these lapses contribute to the observed deficit. Additionally, our findings indicate that the contrast sensitivity deficit in chronic patients may be due to medication rather than the disease itself. In fact, two studies have reported increased contrast sensitivity in first-episode, unmedicated patients. To clarify whether contrast sensitivity could serve as a marker of psychosis, future research should assess this function in both medicated and unmedicated patients, using methods that account for the potential effect of lapses. Furthermore, to clarify the role of spatial frequency and the involvement of the magnocellular system, future studies should measure visual acuity, and address the heteroscedasticity of the contrast sensitivity measures.

## Methods

### Search Strategy

We performed the literature search using the PRISMA guidelines^91^ On Google Scholar and PubMed, we searched the terms *contrast sensitivity schizophrenia*, *contrast threshold schizophrenia* and c*ontrast detection schizophrenia* to identify articles that measured contrast sensitivity in individuals diagnosed with schizophrenia or schizoaffective disorder (search performed on July 11th, 2024). We reviewed the first 10 pages of results from each search engine, with 10 articles per page, yielding a total of 600 references. By reviewing the cited references and tracking subsequent articles citing them, we added 5 additional references to our initial set of articles.

After removing 180 duplicated records, 14 inaccessible records, 8 records in languages other than English, 1 PhD dissertation, 4 abstracts, and 341 that did not measure contrast sensitivity in schizophrenia, we were left with 57 studies. Amongst these,^11,33,36,92–100^ were omitted from our analysis as they involved participants already included in previous studies by the same authors. Therefore, we were left with 46 studies assessing contrast sensitivity in individuals with schizophrenia and healthy controls (Supplementary Table 2). Supplementary Figure 6 shows the PRISMA flow diagram representing the selection process and documenting the number of records identified, included, and excluded at each stage of the review. The three authors established the inclusion criteria and participated in the literature search and screening process.

### Data Extraction

To plot contrast sensitivity across groups for each study (Supplementary Figure 1), we used the reported mean and variability data (standard deviation, confidence interval or standard error) when possible. If those statistics were unavailable, we extracted them from graphs using *Plotdigitizer* (https://plotdigitizer.com/). Supplementary Table 2 indicates the method used for each study. In some cases,^19,66^ the error bars were too small to be visible, so we used the limits of the symbols representing the means. For the study of Serrano-Pedraza and colleagues,^22^ we compute the statistics directly from the raw data sent by the authors. None of the studies made the raw data publicly available except for the study of Zemon and colleagues.^36^ Aster Joostens performed the data extraction from 22 papers, which were subsequently verified by Daniel Linares. The data from the rest of the papers was extracted by Daniel Linares and checked by Cristina de la Malla.

### Characteristics of Participants and Task Parameters

Participants’ demographic and clinical details, along with task parameters are presented in Supplementary Table 2. Next, we provide specific details for some studies. O’Donnell and colleagues 27 measured PANSS in 16 out of 24 patients. Kéri and colleagues^47^ used a score system ranging from 0 to 6 (personal communication) that we adapted to the more conventional 1-7 scale by adding 18 (the total number of items) to the mean BPRS. In Slaghuis and colleagues 5, due to the partial overlap of participants across two experiments, we calculated the weighted average for PANSS, CPZ, and age. In some studies, some participants from the original sample did not have contrast sensitivity measurements. Consequently, the demographic and clinical variables, typically reflective of the entire sample, may present minor discrepancies. For studies where sensitivity data were partially missing, we assumed that half of the excluded participants were female.

Regarding stimulus size, the parameter included in the table was the diameter for circular stimuli, and the side length for square stimuli. For rectangular stimuli, we included the side length of an equivalent square with the same area. Regarding the duration of the stimuli, two studies did not specify the duration as they used the method of limits.^35,45^ For the study of Slaghuis and colleagues,^5^ we included the geometric mean of the durations used in the two experiments as contrast sensitivity was pooled across them. We also used the geometric mean for the durations used in the study of Calderone and colleagues.^58^ Shoshina and colleagues^60^ did not specify the duration. Kantrowitz and colleagues,^67^ Zemon and colleagues^36^ and Qian and colleagues^65^ reported contrast sensitivity for two durations; we selected the longer duration (500 ms) because it was closer to the most common duration used in other studies. Qian and colleagues^65^ reported contrast sensitivity for several luminances; we selected the largest luminance (100 cd/m^2^) because it was closer to the mean luminance used in the other studies (57 cd/m^2^). Maher and colleagues^62^ reported contrast thresholds for faces and trees. However, due to the unavailability of detailed information about the stimuli, we could not determine which stimulus contained more energy near 0.5 c/deg, the spatial frequency most frequently used in other studies. As a result, we made a random choice and selected trees. From Martínez and colleagues^34^ we computed the effect size across spatial frequencies using the F statistic, as the data to compute the effect size for each spatial frequency was not available. Carter and colleagues^23^ used random dots instead of gratings to assess contrast sensitivity. The spatial frequency that we considered in this study was the spatial frequency with more power that resulted from applying Fourier analysis to the stimulus.

For accurate measurement of human contrast sensitivity, it is essential to increase the number of luminance levels beyond the 256 typically displayed by conventional computer monitors, using either hardware or software solutions.^101,102^  Supplementary Table 2 shows the approach used in each study. Among the 46 studies examined, 25 implemented a hardware solution, while none used a software solution. Three studies appeared not to have implemented any specific solution. For 12 studies, the approach was not explicitly mentioned; however, in some cases, based on previous publications by the same authors, it is plausible that some form of solution was indeed used.

### Data Analysis

Given that only two studies reported an effect size based on standardized differences,^29,31^ we estimated Hedges’ g for most studies using the means and standard deviations for each group. This was achieved through the function *esc_mean_sd* from the R Package *esc*.^103^ To estimate the standard deviation from the standard error, we multiplied it by the square root of the sample size. To estimate the standard deviation from the 95% confidence interval, we divided the length of the confidence interval by the difference between the percentiles 75% and 25% of the *t* distribution. In two studies^15,20^ we estimated g from the t statistic using the function *esc_t* from the R package *esc*. In the study of Dias and colleagues,^57^ in which the mean contrast sensitivity for each group was not available, we estimated g from the F statistic using the *esc_f* function from the R package *esc*. Next, we provide specific details for some studies. In the study of Schwartz and colleagues,^35^ we could not estimate the effect size for the static condition due to the absence of the relevant graphs and statistics. This limitation also applied to the data for the spatial frequencies of 7 and 21 c/deg in the study of Revheim and colleagues.^53^ In the study by Kiss and colleagues,^30^ we selected the results from the steady pedestal condition as it was more consistent with the paradigms used in other studies. To assess publication bias, we performed an Egger’s test using the function *eggers.test* from the R package *dmetar*.

In some studies there were two or more groups of patients: with positive- or negative-symptom schizophrenia,^5^ taking typical or atypical antipsychotics,^24,29,50,60^ taking or not taking antidepressants,^18,64^ reading or nonreading impaired,^53^ with or without abnormal neurological signs,^52^ medicated or unmedicated,^27,29^ with or without posttraumatic stress disorder,^21^ tobacco users or nonusers.^63^ In those cases, we combined the effect sizes of the independent samples using the *pool.groups* function from the R package *dmetar*,^104^ applying weighted averages for PANSS, CPZ, and age.

The data and the code to perform the statistical analysis and create the figures is available at github (https://github.com/viscalab/meta_contrast_scz). For the random-effects model, we used the function *metagen* from the R package *meta*^105^ using the restricted maximum likelihood estimator with the Knapp-Hartung adjustments to calculate the confidence interval around the pooled effect.^106^ As measures of heterogeneity, we reported Cochran’s *Q* and Higgins and Thompson’s *I*^2^. To assess the effect of moderators, we performed a meta regression mixed-effects model^75^ using the function *metareg* from the R package *meta*. To perform the three-level meta-analysis^75^ we use the function *rma.mv* from the R package *metafor*^107^ using restricted maximum likelihood.

## Supplementary Material

Supplementary material is available at https://academic.oup.com/schizophreniabulletin/.

sbae194_suppl_Supplementary_Material
