# Supplementary material for: A Systematic Review and Meta-Analysis on Contrast Sensitivity in Schizophrenia
Source: Schizophr Bull. 2024 Nov 22;51(5):1231–41. doi: 10.1093/schbul/sbae194 (PMC12414570; doi:10.1093/schbul/sbae194)
Supplement: sbae194_suppl_Supplementary_Material [file sbae194_suppl_supplementary_material.zip › Supplementary Captions.docx]

*Supplementary Figure 1. Contrast sensitivity as a function of the spatial frequency (x-axis) and temporal frequency in Hz (different panels) in individuals with schizophrenia and healthy controls for each study with available frequencies. A small jitter in spatial frequency between groups is added to minimize overlap. Error bars are standard errors of the mean.*

*Supplementary Figure 2. Hedges' g for each study with available frequencies. Error bars are 95% confidence intervals.*

*Supplementary Figure 3. Temporal and spatial frequencies used in each study with available frequencies. The red dots indicate the frequencies considered for the independent samples meta-analysis.*

*Supplementary Figure 4. Forest plot for all the studies including the weight of each study to the pooled effect size and the sample size.*

*Supplementary Figure 5. Effect of medication on the contrast sensitivity deficit for low and high spatial frequencies. We discretized the frequencies as low and high depending on whether they were smaller or larger than the median (2 c/deg). The slope for low frequencies was 0.0006 (95% CI, -0.0001 to 0.0013; t = 1.8; P = 0.085) and for the high spatial frequencies was -0.0006 (95% CI, -0.001 to 0.0001; t = 1.6; P = 0.11).*

*Supplementary Figure 6. PRISMA 2020 flow diagram for new systematic reviews which included searches of databases and registers only.*

*Supplementary Table 1. Effect of CPZ and the proportion of medicated patients on the contrast sensitivity deficit taking into account all patients (chronic and first episodes).*

*Supplementary Table 2. Demographic and clinical data from the participants and task parameters.*
