# Supplementary material for: A Systematic Review and Meta-Analysis on Contrast Sensitivity in Schizophrenia
Source: Schizophr Bull. 2024 Nov 22;51(5):1231–41. doi: 10.1093/schbul/sbae194 (PMC12414570; doi:10.1093/schbul/sbae194)
Supplement: sbae194_suppl_Supplementary_Material [file sbae194_suppl_supplementary_material.zip › sup_fig_3.pdf]

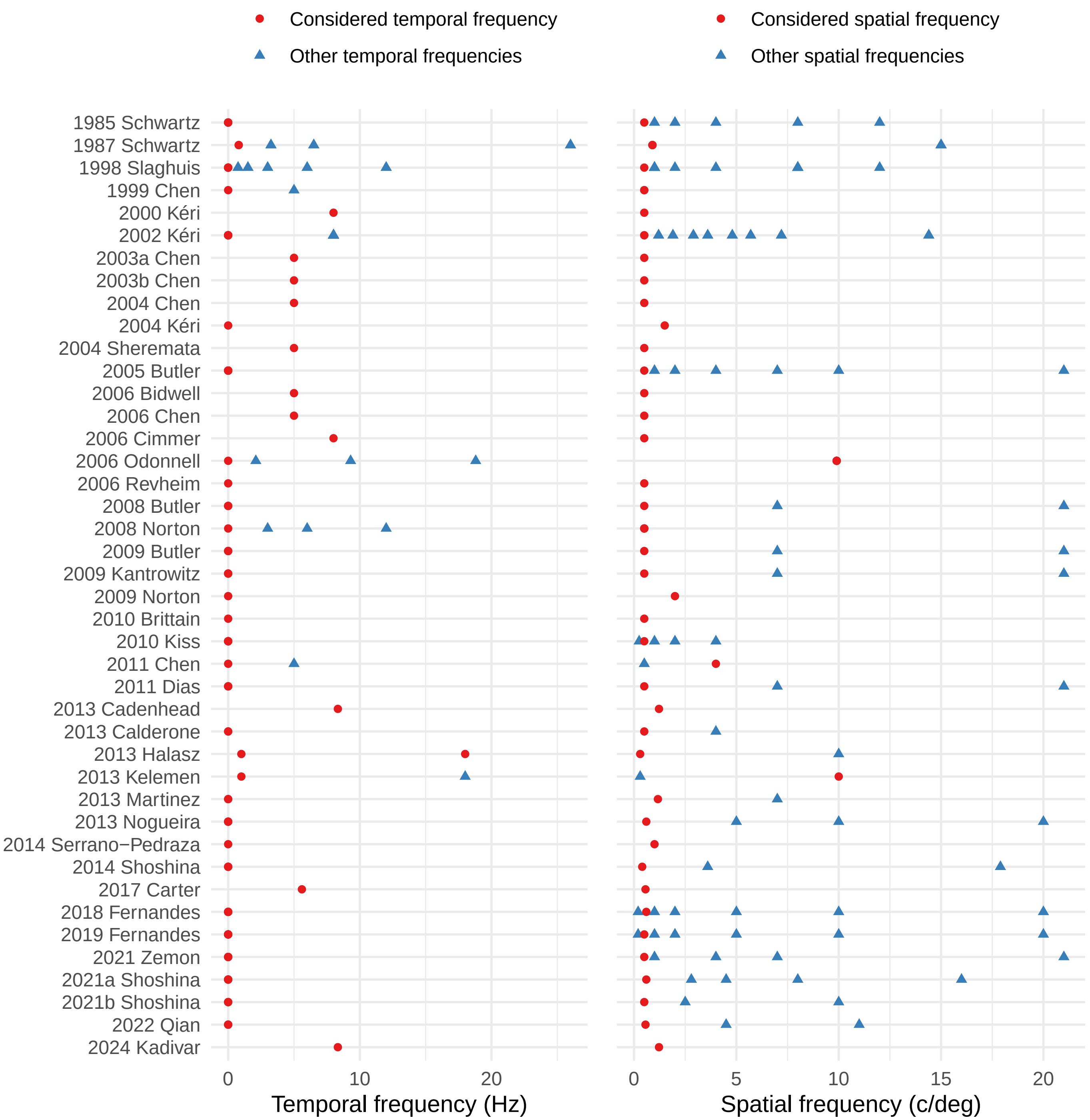

Considered temporal frequency
Other temporal frequencies
Considered spatial frequency
Other spatial frequencies
1985 Schwartz
1987 Schwartz
1998 Slaghuis
1999 Chen
2000 Kéri
2002 Kéri
2003a Chen
2003b Chen
2004 Chen
2004 Kéri
2004 Sheremata
2005 Butler
2006 Bidwell
2006 Chen
2006 Cimmer
2006 Odonnell
2006 Revheim
2008 Butler
2008 Norton
2009 Butler
2009 Kantrowitz
2009 Norton
2010 Brittain
2010 Kiss
2011 Chen
2011 Dias
2013 Cadenhead
2013 Calderone
2013 Halasz
2013 Kelemen
2013 Martinez
2013 Nogueira
2014 Serrano−Pedraza
2014 Shoshina
2017 Carter
2018 Fernandes
2019 Fernandes
2021 Zemon
2021a Shoshina
2021b Shoshina
2022 Qian
2024 Kadivar
0
10
20
Temporal frequency (Hz)
0
5
10
15
20
Spatial frequency (c/deg)
