# Supplementary material for: A Systematic Review and Meta-Analysis on Contrast Sensitivity in Schizophrenia
Source: Schizophr Bull. 2024 Nov 22;51(5):1231–41. doi: 10.1093/schbul/sbae194 (PMC12414570; doi:10.1093/schbul/sbae194)
Supplement: sbae194_suppl_Supplementary_Material [file sbae194_suppl_supplementary_material.zip › sup_fig_6.pdf]

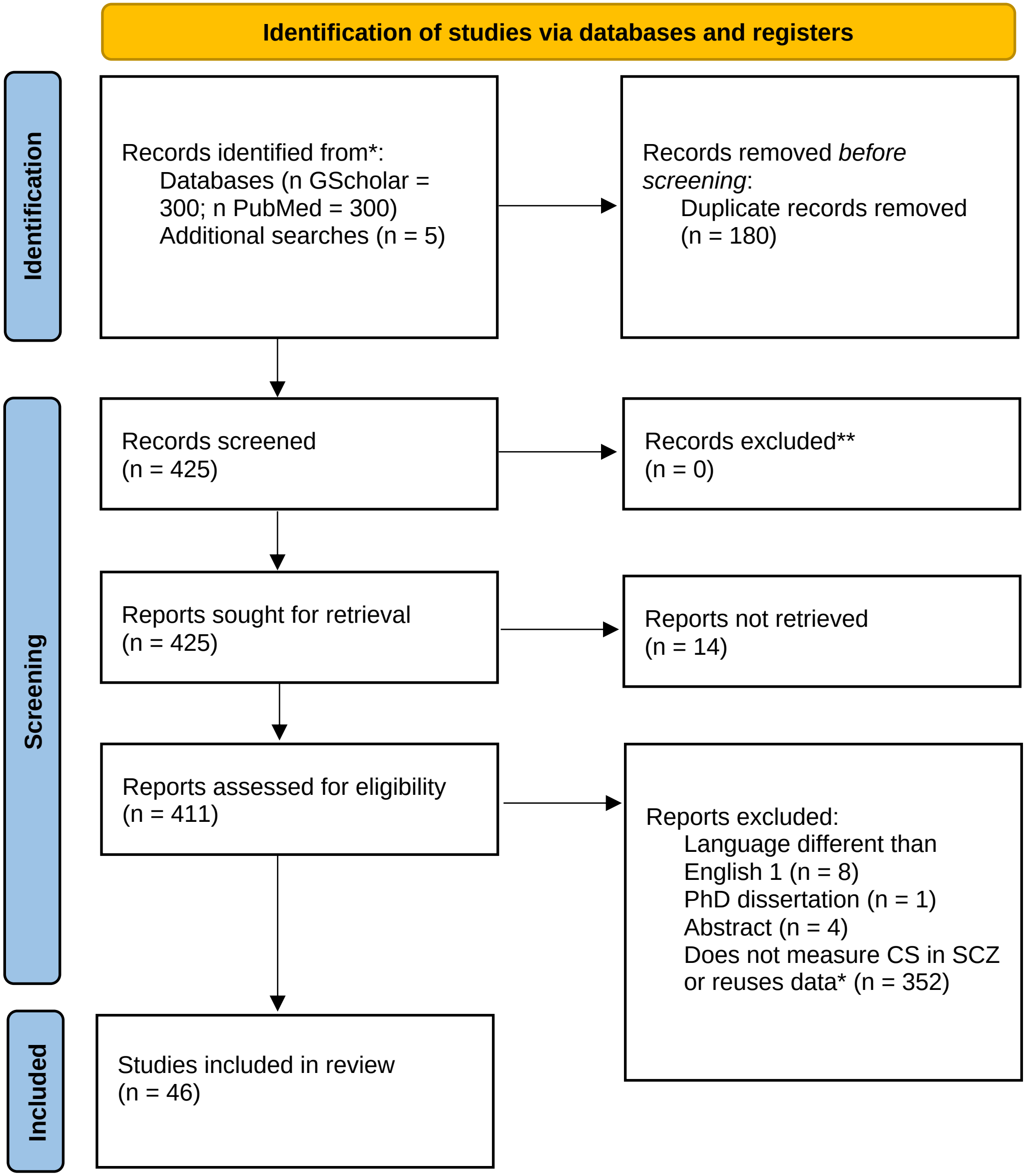

*We considered the articles from the 10 first pages of GScholar and Pubmed for the searches of " contrast sensitivity schizophrenia", "contrast threshold schizophrenia" and "contrast detection schizophrenia". This led to 600 articles. Beyond this, we identified 5 additional articles related to the topic.

**No automation tools were used.

*We excluded reviews and studies measuring contrast surround suppression, contrast in the presence of flankers, or missing a control group. We also excluded studies that reused data from previous experiments.
