# Supplementary figures and images for: A Systematic Review and Meta-Analysis on Contrast Sensitivity in Schizophrenia
Source: Schizophr Bull. 2024 Nov 22;51(5):1231–41. doi: 10.1093/schbul/sbae194 (PMC12414570; doi:10.1093/schbul/sbae194)

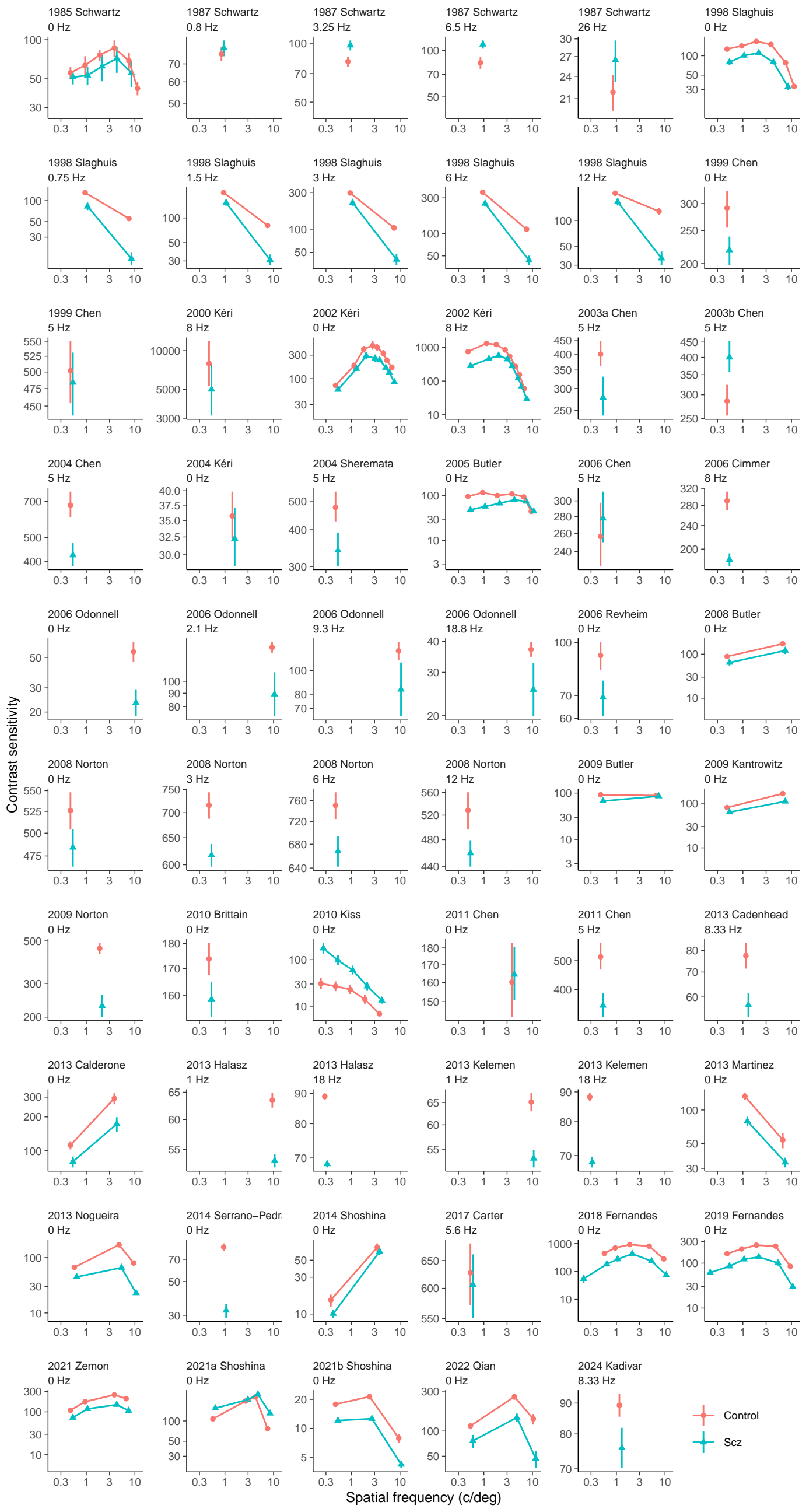

Supplement: sbae194_suppl_Supplementary_Material [file sbae194_suppl_supplementary_material.zip › sup_fig_1.pdf]

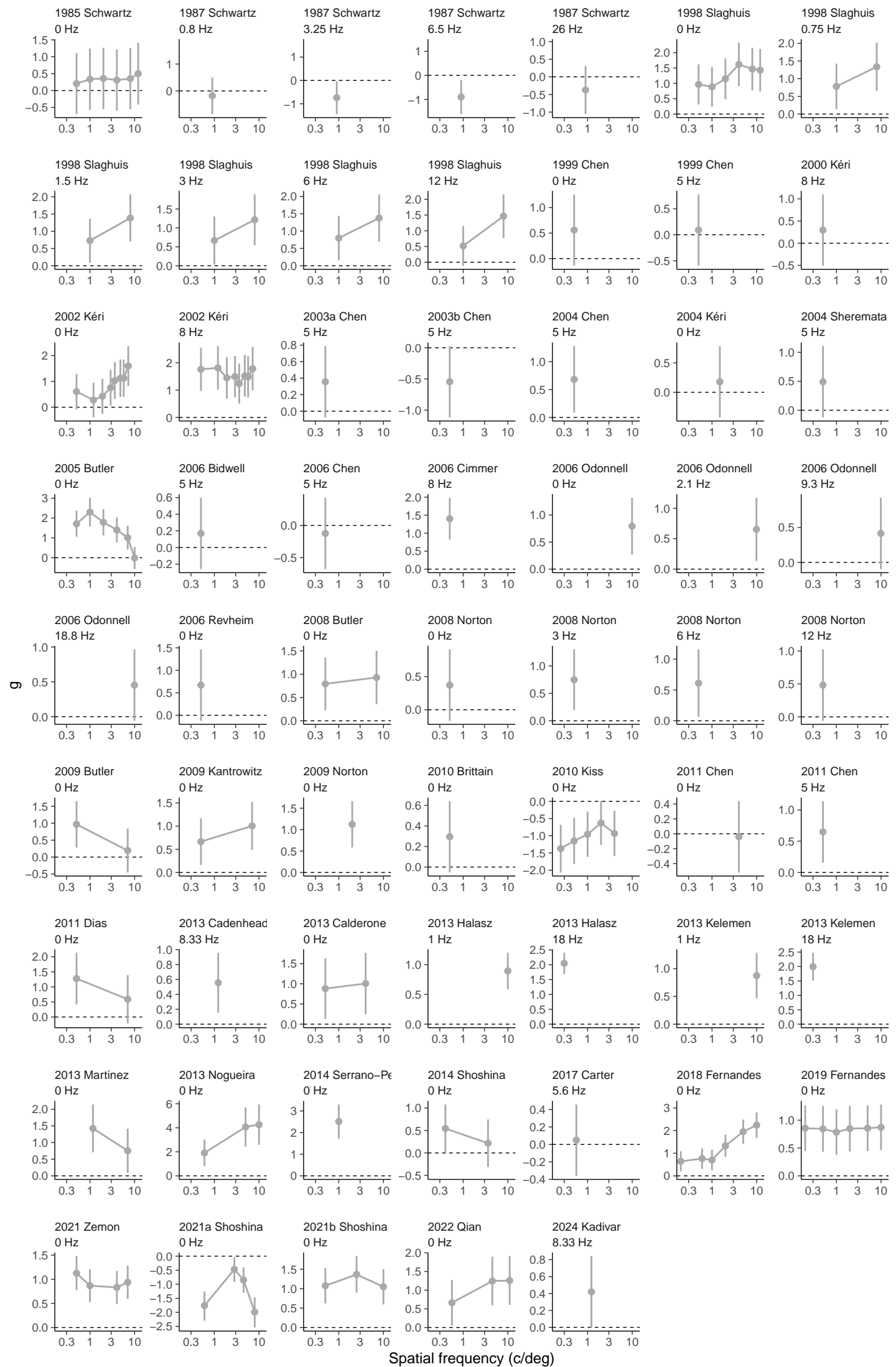

Supplement: sbae194_suppl_Supplementary_Material [file sbae194_suppl_supplementary_material.zip › sup_fig_2.pdf]

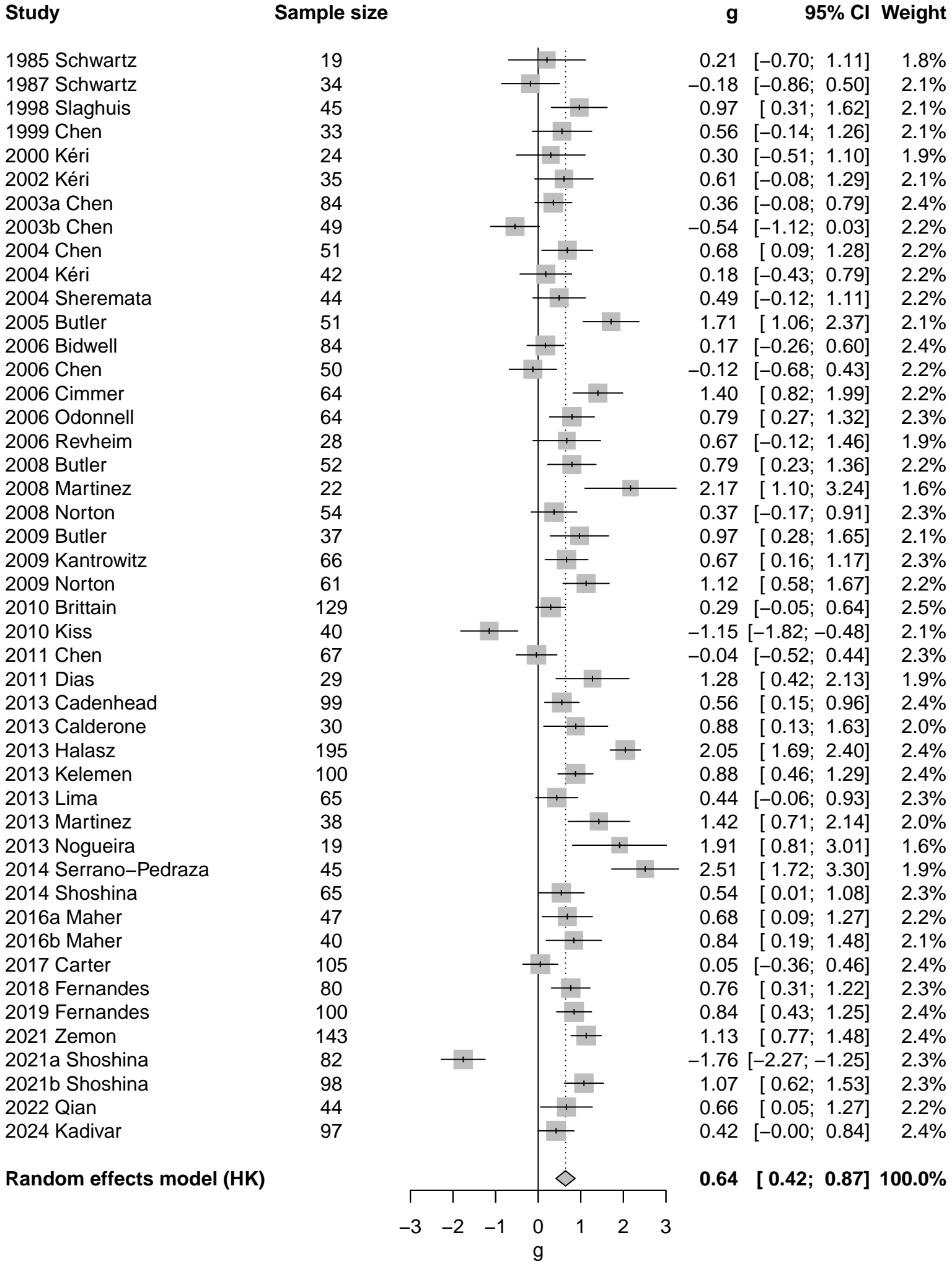

Supplement: sbae194_suppl_Supplementary_Material [file sbae194_suppl_supplementary_material.zip › sup_fig_4.pdf]

Low spatial frequencies

High spatial frequencies

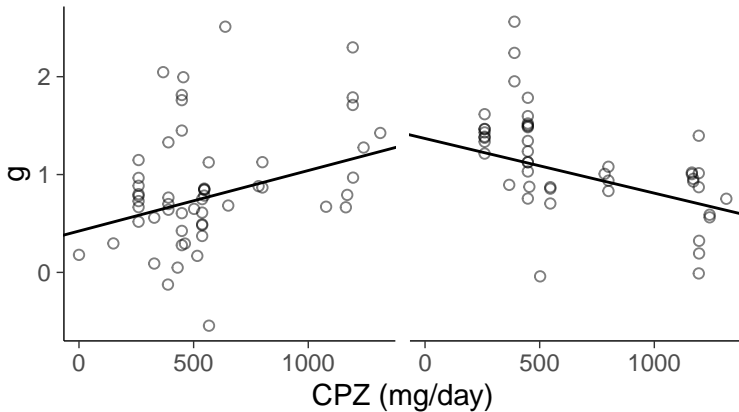

Supplement: sbae194_suppl_Supplementary_Material [file sbae194_suppl_supplementary_material.zip › sup_fig_5.pdf]

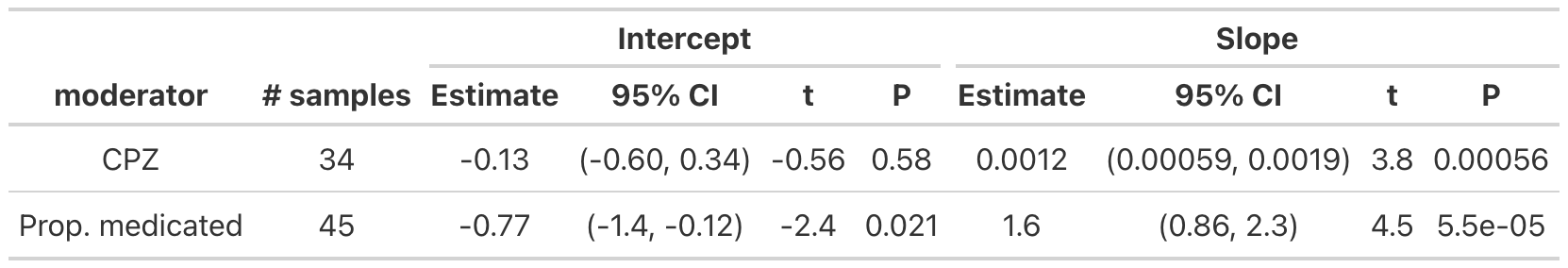

Supplement: sbae194_suppl_Supplementary_Material [file sbae194_suppl_supplementary_material.zip › sup_table_1.png]

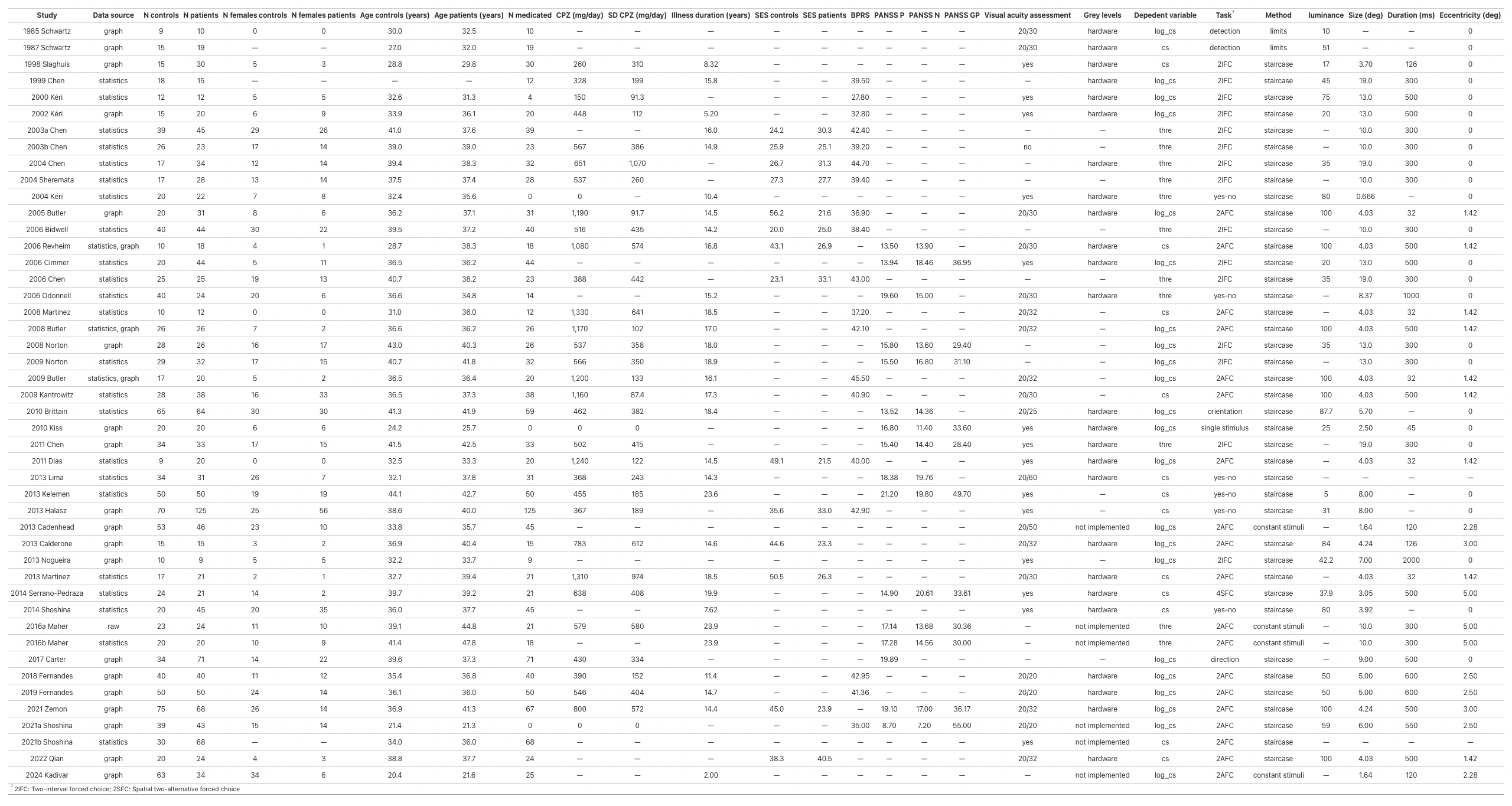

Supplement: sbae194_suppl_Supplementary_Material [file sbae194_suppl_supplementary_material.zip › sup_table_2.png]
